# The Emergence and Early Evolution of Biological Carbon-Fixation

**DOI:** 10.1371/journal.pcbi.1002455

**Published:** 2012-04-19

**Authors:** Rogier Braakman, Eric Smith

**Affiliations:** Santa Fe Institute, Santa Fe, New Mexico, United States of America; JCVI, United States of America

## Abstract

The fixation of 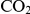 into living matter sustains all life on Earth, and embeds the biosphere within geochemistry. The six known chemical pathways used by extant organisms for this function are recognized to have overlaps, but their evolution is incompletely understood. Here we reconstruct the complete early evolutionary history of biological carbon-fixation, relating all modern pathways to a single ancestral form. We find that innovations in carbon-fixation were the foundation for most major early divergences in the tree of life. These findings are based on a novel method that fully integrates metabolic and phylogenetic constraints. Comparing gene-profiles across the metabolic cores of deep-branching organisms and requiring that they are capable of synthesizing all their biomass components leads to the surprising conclusion that the most common form for deep-branching autotrophic carbon-fixation combines two disconnected sub-networks, each supplying carbon to distinct biomass components. One of these is a linear folate-based pathway of 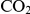 reduction previously only recognized as a fixation route in the complete Wood-Ljungdahl pathway, but which more generally may exclude the final step of synthesizing acetyl-CoA. Using metabolic constraints we then reconstruct a “phylometabolic” tree with a high degree of parsimony that traces the evolution of complete carbon-fixation pathways, and has a clear structure down to the root. This tree requires few instances of lateral gene transfer or convergence, and instead suggests a simple evolutionary dynamic in which all divergences have primary environmental causes. Energy optimization and oxygen toxicity are the two strongest forces of selection. The root of this tree combines the reductive citric acid cycle and the Wood-Ljungdahl pathway into a single connected network. This linked network lacks the selective optimization of modern fixation pathways but its redundancy leads to a more robust topology, making it more plausible than any modern pathway as a primitive universal ancestral form.

## Author Summary

The existence of the biosphere today depends on its capacity to fix inorganic  into living matter. A wide range of evidence also suggests that the earliest life forms on Earth likewise derived their carbon from . From these two observations one can assume that the global biological carbon cycle has always been based on , and we show here that this assumption can be used as a powerful constraint to help organize and explain the deep evolution of life on Earth. Using a novel method that fully integrates aspects of metabolic and phylogenetic analysis, we are able to reconstruct the complete early evolutionary history of biological carbon-fixation, relating all ways in which life today performs this function to a single ancestral form. The diversification in carbon-fixation appears to underpin most of the deepest branches in the tree of life, and this early metabolic diversification – reaching back to the first cells – appears to have been driven not by the contingencies of history, but by direct links to the physical-chemical environment. The ancestral carbon-fixation pathway that we identify is different from any modern form, but better suited to the capabilities of the earliest primitive cells.

## Introduction

Six different autotrophic carbon-fixation pathways have been identified across the tree of life [1]–[3]. It has been recognized that some of these pathways share reaction sequences, but a comprehensive framework does not yet exist to interpret the relatedness among all these extant phenotypes, or to judge which if any is the best candidate for a preserved ancestral form [3]–[6]. Phenotypes exclusive to both the bacterial and archaeal domains have been found, but a full explanation for the patterns of exclusivity has not yet been given [1], [2]. Discussions of the ancestral mode of carbon fixation have focussed primarily on the Wood-Ljungdahl (WL) pathway [7] – as part of the reductive acetyl-CoA pathways – and on the reductive citric acid cycle (rTCA) [8], but diverse observations of metabolic universality and simplicity, network topology, and phylogenetic distribution have not yet been given a single compatible interpretation [3]–[6]. As will be elaborated upon below, throughout this work we use the concepts of carbon-fixation ‘pathways’ and ‘phenotypes’ interchangeably as we focus only on the initial biochemical sequences of how 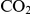 enters living cells.

While consensus remains elusive on which pathway (if any) represents the ancestral form of carbon fixation, it has become increasingly accepted over the last 30 years that the first forms of life were likely chemoautotrophic, deriving all biomass components from 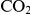, and all energy from inorganic redox couples in the environment [3]–[6], [9]. (Photoautotrophs, in contrast, derive energy from sunlight, while heterotrophs derive energy and biomass from organic sources of carbon.) Most discussions of autotrophy in the origin of life are complicated because they combine chemical requirements for carbon and energy uptake with considerations of whether organisms or syntrophic ecosystems were required to complete the required pathways, and the ways the resulting population processes would have related to genomics on one hand and to metabolic regulations on the other. The disentangling of these issues is addressed in [10], and we will not revisit them here. Instead we focus on the purely biochemical requirements for autotrophy, and adopt the working hypothesis that at the ecosystem level the biosphere has been autotrophic since its inception. Autotrophy provides a simple yet powerful constraint from which we derive a coherent framework for the evolution of all extant fixation pathways.

The modern biosphere may be described, most fundamentally, as implementing a biological carbon cycle based on 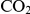, in which carbon fixation is the metabolic anchor embedding life within geochemistry. If the earliest ecosystems were also autotrophic, then a carbon cycle based on 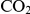 must have existed continuously to have supported biosynthesis. Any local ecosystems that could no longer fix 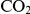 either would have become appendages to neighboring autotrophic ecosystems, or would have dwindled and been recolonized by such ecosystems. Under either scenario it must be possible, relative to appropriately defined ecosystem boundaries, to trace all extant carbon-fixation pathways through fully autotrophic sequences to the earliest forms. The question for historical reconstruction is then whether these sequences are sufficiently simple that their innovations could have occurred independently, or whether complex, cross-species and interdependent sequences of innovations sustained continuous carbon-fixation. In the latter case ecosystems become the relevant units of phylogenetic comparison, and clear lineages in carbon-fixation may be difficult, if not impossible, to reconstruct. A history of independent innovations, in contrast, allows us to be indifferent to the distinction of ecosystems and species, and should make clear carbon-fixation lineages distinguishable.

In this paper we reconstruct the evolutionary sequences that relate modern carbon-fixation pathways to each other and to a single ancestral form, showing that the history of carbon-fixation can indeed be described as simple sequences of independent innovations in autotrophy. To define the constraint of autotrophy we will use metabolic-flux balance analysis, and because we do not use it to model cellular-level mechanisms of either regulation or heredity, it does not distinguish among strictly defined autotrophic species, populations of diverse cells (or pre-cells) tightly linked by transfer of genes and metabolites [11], [12], or syntrophic ecosystems. Therefore the ‘carbon-fixation phenotypes’ that we analyze may, but need not, have corresponded to phenotypes integrated within single species. The intensity of gene transfer and the integrity of species lineages thus become moot points, unless they lead to signatures of complexity and non-independence in the sequence of carbon-fixation phenotypes. Moreover, we focus here on the integrated pathways of carbon fixation alone, requiring only that in the bottom-up construction of biomass all initial metabolic branching points be directly accessible from 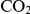, and do not extend to the more complicated reconstruction of full intermediary metabolism. The remarkble modularity and redundancy of carbon fixation pathways, and the small number of metabolites through which they are connected to the rest of metabolism, make this separation feasible for autotrophy. We return to the complexities that arise upon consideration of heterotrophic organisms and larger components of intermediary metabolism in a later section. We thus describe a diversification of the of input channels of carbon into the biosphere, from which downstream anabolic pathways may diverge in different organisms. (Anabolism is the process by which life constructs larger organic metabolites from smaller ones, while catabolism is the converse breakdown of larger into smaller molecules.) Distinctions caused by the participation of heterotrophs in complex ecosystems, as well as those among organisms that share carbon-fixation pathways but use these to feed diversified intermediary metabolism, all arise outside the networks we model here. The ancestral pathways we reconstruct are therefore best understood as divergences in the ecosystem-level metabolic foundation of an emerging biosphere. Our evolutionary reconstructions are based on a novel integrated metabolomic/phylogenomics approach, whose basic principles we outline next.

## Results/Discussion

### Integrating phylogenetic and metabolic analyses

The two main statistical tools that exist to probe genetic information and study the early metabolic evolution of life are phylogenetics [13]–[15] and metabolic flux-balance analysis (FBA) [16], [17]. Whole-genome FBA models have become a widely used and successful method to study the metabolism of individual organisms [16], [18]. One can use this approach and target deep-branching organisms in an attempt to study conserved metabolic features near the base of the tree of life. However, due to the inherent ambiguities and errors in using gene sequence comparisons to determine the presence of enzymes [19], [20] and therefore reactions, heuristics are needed to fill the “gaps” in the initial network that is derived from the genome to produce a viable organism model [16], [18]. These techniques work remarkably well in predicting overall growth rate dynamics under various conditions, especially for well-characterized model organisms [16], [18]. It is less clear, however, what confidence to assign to such heuristic rules when targeting individual pathways of evolutionary interest, especially for deep-branching organisms that lack extensive laboratory characterization and that have significant gene divergences from well-characterized model organisms. Similarly, phylogenetics based on gene presence/absence and sequence similarity, without other prior constraints, has given significant insights into the relatedness and historical divergences among organisms [13], [14]. However, branching relationships become more ambiguous at greater phylogenetic depths, as tree-like descriptions fail for whole organisms due to extensive lateral gene transfer (LGT) near the root [12], [14], [21]. This can make the phylogenetic position (and evolutionary divergence) of metabolic pathways uncertain, as has been the case for carbon-fixation pathways [1], [2].

**Figure 1:**
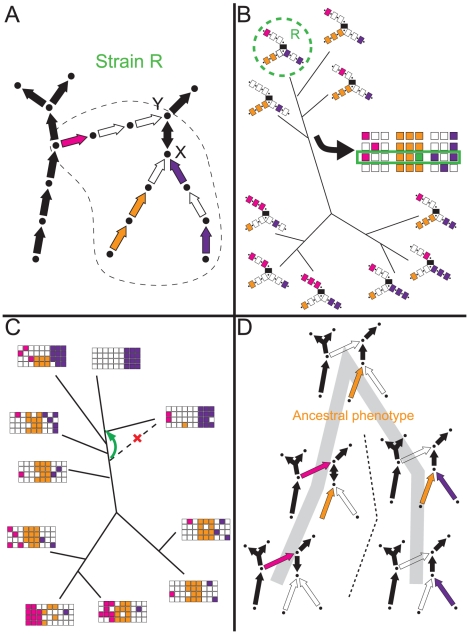
Principles of phylometabolic analysis. **A** - *Example metabolic network structure*. Two essential metabolites, X and Y, can be synthesized through three known metabolic pathways, indicated by magenta, orange and purple arrows. Individual arrows represent enzymes catalyzing different steps in the pathway, and they can be either absent (empty arrows) or present (filled arrows) in the annotated genome of a particular strain R. Arrows in black are universal metabolic genes. **B** - *Phylogenetics constrains metabolic analysis*. Each branch in this phylogenetic tree represents an individual strain. The gap structure in the metabolic network of strain R, which was difficult to interpret in isolation becomes much less uncertain when placed in a phylogenetic context, suggesting that in this organism the orange pathway should be completed. **C** - *Metabolic analysis constrains phylogenetics*. Each branch in this tree represents a clade and individual (horizontal) rows within the metabolic gene profile matrices represent the profile of individual strains. Here the metabolic context suggests proper placement of a clade-level branch that was uncertain in an unconstrained phylogenetic tree. **D** - *Fully integrated phylometabolic tree*. Metabolic sequences are now drawn at the pathway level, and this tree represents a reconstruction of the evolution of the synthesis pathways of metabolites X and Y. Because mechanisms of regulation or heredity are not modeled, this phylometabolic tree is indifferent to distinctions of ecosystems or species, and the shown ‘phenotypes’ refer exclusively to the biochemistry of these pathways (see text for further details). Fig. 1C suggested that the orange pathway was ancestral, and that the magenta and purple pathways were derived from it. The only sequences that are then allowed under the constraint that essential metabolites X and Y are continuously produced, is the appearance of the purple and magenta pathways from stages in which they are co-present with the orange pathway.

Phylogenetics and flux-balance analyses, if used together, have complementary strengths that may ameliorate some of these problems. We refer to the joint use of the two methods as “phylometabolic” analysis, and illustrate its main features in Fig. 1. Instead of using heuristics to fill gaps in order to complete an initial network derived from genome annotation (as for example in Fig. 1A), we compare the gene profile for a metabolic pathway in a focal organism to those in related organisms both within and across neighboring clades as shown in Fig. 1B. By focusing at the pathway level, the comparison may reveal variations in multi-genic functional units, providing context for the completion of individual organism networks, while also restricting the plausible sequences for divergence in the evolution of metabolic structure. Especially in cases where individual reactions or growth conditions, rather than complete pathways, have been chemically characterized in different organisms, comparison of functional units can pool evidence that would not be restrictive in isolation. Conversely, as shown in Fig. 1C, placing hypothesized constraints on functional units, such as requiring the continuous production of essential metabolites, can lead to specific conclusions about uncertain clade-level branches in phylogenetic trees. This may be understood as adding a semantic dimension for the contribution of genes to phenotypes, which provides a different form of disambiguation, when phylogenies based on gene presence/absence alone yield poorly defined deep branching relationships as summary statistics for reticulated networks of single-gene histories [14], [21]. The reconstructed “phylometabolic tree”, shown in Fig. 1D, by including multiple complete pathways to common essential metabolites, then suggests which evolutionary substitutions are allowed (at either organism or ecosystem levels) among these pathways by the constraint that essential metabolites are continuously produced. As we will show for the evolution of carbon-fixation, complete pathway reconstructions, combined with characterization of reaction mechanisms and enzymes, often suggests the causes for reconstructed divergences. In an analysis of large or highly ambiguous networks, statistical methods already employed for gene phylogenies, or those used to suggest enzyme functions in automated FBA modeling, could be combined into joint-maximum-likelihood or Bayesian Markov Chain Monte Carlo (MCMC) reconstruction algorithms. The small size and high parsimony of the network we will consider permitted manual reconstruction.

### Phylometabolic analysis of carbon-fixation: general findings

**Figure 2:**
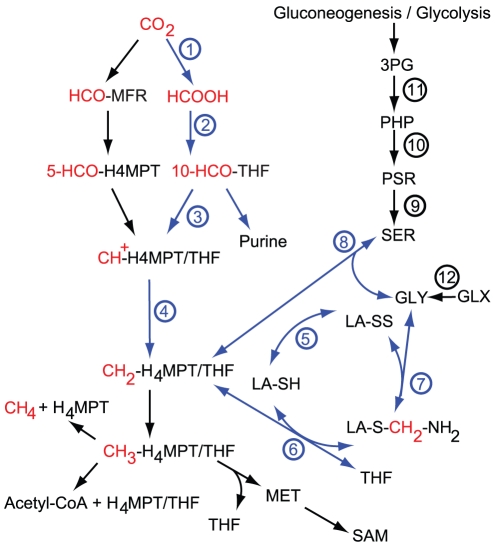
Summary of 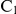 metabolism. Reactions 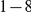 (highlighted in blue) represent, as we show here, the ancestral route to glycine and serine. In organisms that use the Wood-Ljungdahl pathway as the exclusive route to carbon-fixation, the direct reduction of 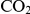 also culminates in the synthesis of acetyl-CoA, a step absent in many other organisms that do employ the preceding parts of the pathway. Reactions 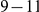 constitute the oxidative route to serine and glycine, which are subsequently cleaved to supply 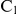 metabolism in many late-branching bacteria and eukaryotes. Reaction 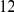 constitutes the glyoxylate pathways, important in cyanobacteria and photorespiring plants. Abbreviations: MFR, methanofuran; 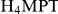, tetrahydromethanopterin; THF, tetrahydrofolate; MET, methionine; LA, lipoic acid; GLY, glycine; SER, serine; PSR, phosphoserine; PHP, 3-phosphohydroxypyruvate; 3PG, 3-phosphoglycerate; GLX, glyoxylate. For a more detailed figure and caption, as well as names and EC classes of numbered reactions see Text S1 and Fig. S1 in Text S1.

Future work will extend these methods to the reconstruction of full models of intermediary metabolism – in a separate manuscript we will describe the metabolic reconstruction of the deep-branching hyperthermophilic chemoautotroph *Aquifex aeolicus*. For the carbon-fixation networks considered here, important interactions of phylogenetic and flux-balance constraints occur at two distinct points in the network. The first, of the kind shown in Fig. 1B, determines our reconstruction of the ancestral synthesis route to glycine and serine, which from the perspective of 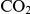 input form a unique set among the monomer precursors to biomass (*e.g.* amino acids, nucleotides). It has been observed [22] that all anabolic pathways originate from five universal precursors: acetyl-CoA, pyruvate, oxaloacetate, succinyl-CoA and 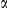-ketoglutarate, and that all of these are intermediates in the citric acid (TCA) cycle. Even in organisms whose carbon-fixation pathways do not pass through this cycle, TCA arcs are used as supplementary pathways to connect primary carbon inputs to these standard precursors. The sole exceptions are glycine and serine (and a few higher order derivatives of these), which, as shown in Fig. 2, can in some organisms be produced directly from 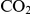, bypassing both the TCA intermediates and all of the recognized complete carbon-fixation pathways. Operation in the fully reductive direction of the folate-based chemistry that forms the core of this pathway has only been observed within the context of the complete Wood-Ljungdahl pathway, in which acetyl-CoA is synthesized as the final step. However, all steps in this sequence to glycine and serine are fully reversible [23], [24], and so there is no reason *a priori* to exclude the possibility of much wider use of this pathway in the context of carbon-fixation.

In the next section we will present evidence strongly suggesting that this pathway is in fact widely present across the tree of life without the final step to acetyl-CoA in organisms that lack alternate routes to glycine and serine, and that it forms the most likely ancestral pathway to these amino acids. This is surprising, not only because carbon fixation on folates had previously only been recognized in anaerobic organisms using it as the sole route to fix 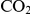, but also because our results suggests that organisms commonly use two disjoint parallel pathways to fix carbon. The presence of two parallel carbon-fixation pathways in an autotroph was only recently noted in a single, late branching 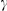-Proteobacterium [25] (begging the question how common this might be [2]) but even in that case the two pathways are connected through metabolic intermediates. We will show evidence that direct reductive synthesis of glycine and serine combines with all other carbon-fixation pathways, and that in many of these cases the two pathways supply distinct, disconnected components of biomass.

Perhaps the most important consequence of these findings is that it significantly increases the similarity among all carbon-fixation phenotypes. In particular, the most similar carbon-fixation phenotypes are now the deep branching form of rTCA, which combines a complete rTCA cycle with an incomplete WL pathway to synthesize glycine and serine, and Wood-Ljungdahl, which combines a complete direct reductive sequence from 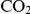 to acetyl-CoA with partial TCA cycle sequences to complete the set of universal anabolic precursors. Moreover, among all carbon-fixation machinery, the folate-based direct reduction of 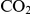 and partial TCA sequences appear to have the most universal distributions across the tree of life, supporting an early appearance for both. Together these various observations suggest general principles underlying the evolutionary diversification of carbon-fixation, as well as an avenue to the reconciliation of phylogenetic and metabolic observations that previously appeared incompatible.

The elaboration of these results below will lead to us to a second junction at which we will invoke phylometabolic constraints, this time of the type in Fig. 1 C/D, in reconstructing the complete evolutionary sequences that connect all carbon-fixation phenotypes to a single ancestral form. As we go through these analyses we also make observations on the nature of the associated reactions and enzymes of key steps in the different forms of carbon-fixation, from which we identify plausible ecological and evolutionary explanations for the divergences at different stages. In particular we include a section immediately following the analysis of glycine/serine pathways that describes in detail how one of the major driving forces - energy optimization - is inferred from a wide range of evidence. The discussion of this driving force, and its interaction with others, will then set the stage for interpreting the full evolutionary history of carbon-fixation. We begin, however, by describing direct reduction of one-carbon (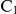) units in more detail, which is not only an essential source of core metabolites in all WL organisms, but occurs ubiquitously as a module for carbon fixation in interaction with other autotrophic pathways.

### Direct one-carbon reduction and hybrid carbon-fixation

Direct 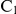 reduction follows the ‘central superhighway’ of tetrahydrofolate (THF) metabolism (some archaea use tetrahydromethanopterin (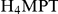) and related compounds, all analogues of THF [24]), which links the synthesis of purines, thymidilate, formyl-tRNA, serine, and methyl-group chemistry mediated by S-adenosyl methionine (SAM) [24]. The core reactions of this pathway, summarized in Fig. 2, are widespread in both oxidative and reductive form throughout the tree of life [24], [26]. In their reductive form these reactions, followed by the acetyl-CoA synthase reaction – catalyzed by one of the most oxygen-sensitive enzymes in our biosphere [27] – make up the WL pathway, which is the principal carbon fixation route in a variety of bacteria and archaea, including acetogens and methanogens, sulfate reducers, and possibly anaerobic ammonium oxidizers [2], [28]. In autotrophs, the WL pathway couples in a variety of ways to an incomplete rTCA cycle forming what are collectively known as reductive acetyl-CoA pathways. We will present evidence from genome comparisons that direct 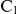 reduction is not only a carbon fixation route in WL organisms, but that it is ubiquitous, and was actually the ancestral route to glycine and serine, which took on diversified roles independent from the complete WL pathway very early in the rise of oxygen. The extant glycine and serine synthetic pathways provide the key constraints in reconstructing ancestral carbon fixation, so we describe these next. From their phylogenetic distribution, and their energetics in the context of fully autotrophic networks, we then reconstruct a sequence for the major innovations in carbon fixation.

Three main synthetic pathways to glycine and serine are found in modern organisms and provide evidence about ancestral forms. In eukaryotes and most late-branching bacteria, serine is derived from 3-phosphoglycerate (3-PG) as a branch from either glycolysis or gluconeogenesis [26], [29], through reactions 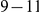 of Fig. 2. Serine is subsequently cleaved to glycine, donating a methylene group to THF, and glycine can be further broken down in what is known as the glycine cleavage system to 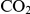 and 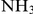, donating a second methylene group to THF [23]. We will refer to this route as the “oxidative” pathway because the first step from 3-PG involves the oxidation of the alpha-hydroxyl to a carbonyl group, and parts of the THF pathway operate in the oxidative direction. In the alternative, direct synthesis of glycine from 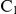 units (reactions 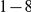 in Fig. 2), the THF-mediated reactions proceed exclusively in the reductive direction, and we refer to this route as the “reductive” pathway. The third major route to glycine is *via* reductive transamination of glyoxylate [29] (reaction 12 in Fig. 2), which is important in cyanobacteria and plants undergoing photorespiration [30]. Following its synthesis from glyoxylate, one molecule of glycine may be cleaved to 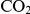 and 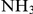, donating a methylene group to THF, which is then combined with a second glycine to produce serine. In photosynthetic organisms, glyoxylate arises from the Calvin-Benson-Bassham (CBB) cycle upon exposure to oxygen [30], but in other organisms it can arise from other sources such as cleavage of isocitrate in the glyoxylate shunt [29]. We track the distribution of glyoxylate transaminase in our gene profile comparisons as the key reaction in all these pathways, and refer to them collectively as the “glyoxylate” pathways. The synthesis of glycine through cleavage of Threonine has been shown to play a role in some organisms (notably *Saccharomyces cerevisiae* [31], [32]), but its physiological importance is generally not well understood [33], [34]. We interpret this route as a salvage pathway and do not consider it further here.

**Figure 3:**
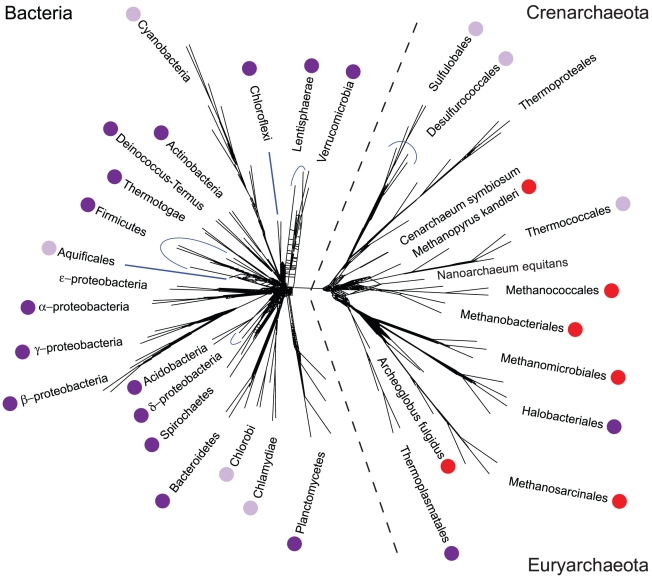
Phylogenetic distribution of direct 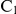 pathways in Archaea and Bacteria. Dark purple dots indicate clades in which the complete gene complement for direct reductive synthesis of glycine and serine is found, light purple dots indicate clades where a complete pathway is suspected. Red dots indicate clades in which direct reduction of 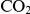 is known to be active, but in a form that lacks synthesis of glycine and serine. See main text for further details. The unrooted phylogenetic tree was adapted, with modification, from [14]. In that work, the tree was created from an analysis of 102 ‘nearly universal’ clusters of orthologous groups of proteins (COGs) (present in 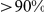 of species in the tree) [14].

To understand the distribution and reconstruct the history of glycine and serine synthesis, we acquired gene profiles for all three pathways from the UNIPROT database [35], for all strains in a wide range of deep-branching organisms (see Methods for details). UNIPROT derives from the manually reviewed SWISSPROT database, which has one of the lowest rates of mis-annotation among public databases [20]. We find the complete gene complement for the reductive pathway widely distributed among both bacteria and archaea, as shown in Fig. 3, including many non-WL organisms that lack the genes for alternative routes. This latter group includes members of clades that use the rTCA cycle (the Nitrospirae) [36] or the 3-Hydroxypropionate (3-HP) bicycle (the Chloroflexi) [37], anaerobic and aerobic heterotrophs (the Thermotogae and *Isosphaera pallida* of the Planctomycetes) [38], [39], and also several archaea (all listed in Table S1 in Text S1). The very wide distribution of direct 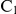 reduction on THF suggests that hybrid carbon fixation is much more common than has been recognized, and that the reductive pathway is not limited to WL organisms in which it is the sole pathway. Indeed, this pathway appears to be more widely distributed than any single primary fixation pathway.

A detailed distribution of gene profiles for the three pathways is shown in Table 1. Although the reductive pathway requires 7 or 8 steps (heterotrophic from formate or autotrophic from 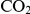, respectively) to reach glycine, versus 3 and 1 in the alternative pathways, its frequency in the sample is nearly double the combined frequencies of the oxidative and glyoxylate pathways.

**Table 1:** Complete Glycine/Serine synthesis pathways in deep-branching bacteria and archaea.

	Pathway					
Bacteria	Glx^a^	Ox^b^	Red^c^	Red(-2)^d^	GlyC^e^	FDH^f^
Aquificae (7)	0	0	0	3	3	5
Thermotogae (11)	0	0	11	0	11	1
Firmicutes (257)						
*Bacilli - Bacillales* (90)	1	13	71	18	89	66
*Bacilli - Lactobacillales* (90)	0	16	0	0	0	5
*Clostridia* (77)	6	6	37	1	39	27
Deinococcus-Thermus (9)	0	0	2	6	8	5
Chloroflexi (14)	0	2	8	0	8	9
Chlorobi (11)	0	0	0	9	11	2
Planctomycetes (4)	1	1	1	3	4	2
Nitrospirae (2)	0	0	1	0	1	2
Verrucomicrobia (4)	1	1	4	0	4	3
Cyanobacteria (40)	22	1	0	38	39	4
All (359)	31	40	135	78	217	131

Archaea	Glx	Ox	Red	Red(-2)	GlyC	FDH
Thermoproteales (8)	2	1	0	0	0	7
Desulfurococcales (8)	2	0	0	0	5^g^	3
Acidilobales (1)	0	0	0	1^g^	1^g^	1
Sulfulobales (12)	1	0	0	0	12^g^	12
Marine I (2)	1	0	0	0	0	0
Korarcheota (1)	0	0	0	0	1^g^	0
Aciduliprofundum (1)	0	0	1^g^	0	1^g^	1
Thermococcales (7)	0	0	0	0	7^g^	7
Thermoplasmatales (3)	0	0	3	0	3	2
Halobacteria (13)	6	0	3	7	11	8
Methanococcales (12)	3	1	0	0	0	10
Methanobacteriales (6)	0	1	0	0	0	6
Methanopyrus (1)	0	0	0	0	0	0
Archeoglobus (3)	0	0	0	0	0	2
Methanosarcinales (6)	0	3	0	0	0	6
Methanomicrobiales (8)	1	2	0	0	0	8
All (92)	16	8	7	8	41	73

Notes:

*^a^*Glx = Glyoxylate pathways (rxn 12),

*^b^*Ox = Oxidative pathway (rxns 9–11),

*^c^*Red = Reductive pathway (rxns 1–8),

*^d^*Red(-2) = Reductive pathway missing only rxn 2,

*^e^*GlyC = Glycine cycle (rxns 5–8),

*^f^*FDH = Formate dehydrogenase (rxn 1),

*^g^*18 archaeal strains that lack only reaction 5 in the glycine cycle were included. For 7 such sulfulobales and the acidilobale a BLASTp search finds a protein that closely matches to this protein in strains not missing it.

The reductive pathway from 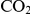 appears in two common forms. The full pathway (“Red” in the tables) comprises 8 reactions. In the alternative form (denoted “Red(-2)”), reaction 2, attaching formate to THF, does not appear in genome searches based on sequence similarity, but *all* 7 other reactions are present. We will argue from detailed analysis of gene frequencies and growth conditions, that the 7-reaction form is active and is in fact a carbon fixation pathway, suggesting that formate incorporation is catalyzed by an unrecognized protein or an unidentified function among the known THF-interconversion enzymes. We suspect that an alternate route involving incorporation through 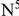 rather than 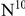 of THF may be active in these cases (see Text S1 for further details). In Table 1 pathways from 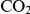 (autotrophic form) and formate (heterotrophic form) are combined under columns Red and Red(-2). For full 8-step autotrophic gene profile see e.g. organisms MTA, CAG and NDE in Table S1 in Text S1. For full alternate 7-reaction autotrophic gene profiles see e.g. organisms AAE, CCH or NPU in Table S1 in Text S1. Full organism names are also found in Text S1. The most informative distributions come from three clades that are consistently placed among the deepest-branching bacteria: the Firmicutes, Thermotogales and Aquificales [40]–[42]. Thermotogales, Aquificales, and several groups of Firmicutes are among the most thermophilic bacteria and are generally restricted to hydrothermal vents [7], [38], [42], [43]. These environments are among the least changed from early-Earth conditions, and clades apparently restricted to them throughout history may well be the most conservative of metabolic features from the base of the tree [44].

Among the Firmicutes, a remarkably diversified clade, the reductive pathway is the common form, the oxidative pathway is less common, and glyoxylate pathways are very rarely found. The only systematic exception to the common pattern among Firmicutes is found in the Lactobacillales, where the ‘glycine cycle’ (reactions 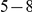 in Fig. 2) is completely absent, apparently having been replaced by the oxidative pathway. Indeed, this group shows the most complete such replacement found among deep-branching bacterial clades. The Lactobacillales, however, are mesophiles, highly adapted to environments rich in organic carbon, and are known to have a high degree of associated gene loss and acquisition through LGT [45]. Therefore we conclude that the reductive pathway is the ancestral route to glycine and serine in the Firmicutes.

The Thermotogales and Aquificales are much less diverse than the Firmicutes, comprising almost exclusively hydrothermal vent/spring organisms. Metagenomic evidence suggests the existence of specialized mesophilic Thermotogales [46], [47], but the amino acid composition of reconstructed ancestral states of a large number of gene families supports a highly thermophilic origin for this clade as a whole [42]. Mesophilic Thermotogales have not yet been cultured and our genomic sample includes thermophiles only. As can be seen, all Thermotogales and Aquificales lack two, or even all three, of the genes to synthesize serine from 3-PG, and none has the gene for glyoxylate transaminase. As the alternative, *all* Thermotogales have a complete reductive pathway, while several Aquificales show the 7-reaction sequence missing only the ATP-dependent formyl-THF synthase (reaction 2). Evidence that formate is, nevertheless, taken up in the reductive pathway by Aquificales comes from the presence of a formate dehydrogenase (reaction 1) that, in obligate autotrophic members of this clade, has been shown *not* to operate in the oxidative direction [48], [49]. Experiments in [48] followed the protocol of [49] that test only for the oxidative direction and found no activity in obligate autotrophs. Hence, this enzyme likely functions as a 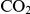 reductase rather than a dehydrogenase, despite the gene nomenclature.

In the remaining deep bacterial lineages, oxidative and reductive pathways are co-present, although the reductive pathways remain more common. The glyoxylate pathway is common only in cyanobacteria (for reasons explained below), and otherwise only in the Halobacteria, a late-branching mesophilic *archaeal* clade restricted to hypersaline environments. Therefore all distributional evidence suggests that not only is the (either latent or active) reductive pathway common across the bacterial domain, but also that it represents the ancestral pathway to glycine and serine. This leads to the general conclusion that the ancestral function of THF-based chemistry was completely reductive starting from 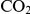, until alternative routes to glycine and serine became available and parts of this chemistry could reverse direction.

**Figure 4:**
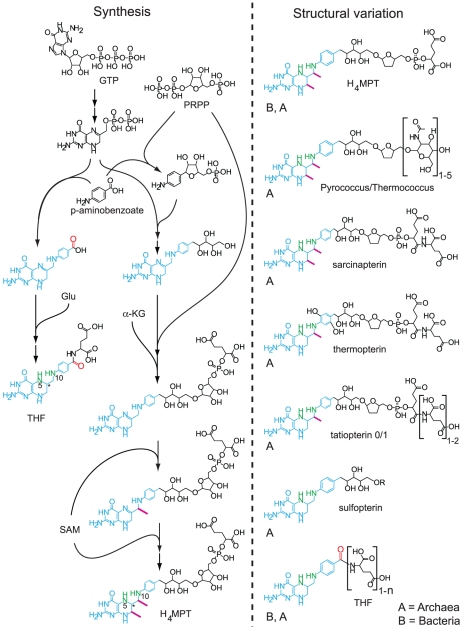
Synthesis of and structural variation in the folate family. The sequences on the left show how the key reactions in the synthesis of tetrahydrofolate (THF) and tetrahydromethanopterin (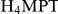) diverge starting from GTP. The structures on the right show the observed variants within the modified folate family and the domain within which they are observed. Unlike other pterins, those observed within Pyrococcus and Thermococcus were not named, hence the name of the clades containing them is shown instead. Within the chemical structures, the core structure that is universal across the folate family, and derived from GTP and aminobenzoate, is highlighted in blue. Numbered and highlighted in green are the 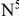 and 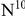 positions of the folates, upon which chemical processing of 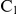 units occurs (see Fig. S1 in Text S1). The carbon highlighted with an asterisk (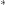) has the same chirality upon reduction in the final step in both THF and 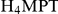 synthesis, further highlighting their relatedness. Highlighted in red is the carbonyl group resultant from aminobenzoate that is present in THF but absent in all modified folates, resulting in a large difference in electron density at 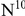 between these two groups. Highlighted in purple are structural methyl groups, which are absent in THF but present in 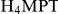. The modified folates as a group show variation in the number of methyl groups they contain in the same order in which they are added in the synthesis of 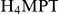. Abbreviations: PRPP, phosphoribosyl pyrophosphate; Glu, Glutamate; 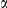-KG, 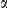-ketoglutarate; SAM, S-adenosyl methionine. The information used to create this figure draws on results from a wide range of experimental studies, including on the synthesis of THF and 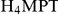 [55]–[58], and on modified folates found in the archaeal domain [59]–[64].

The pattern of pathways for glycine synthesis in archaea is more complex than in bacteria, because pterin diversity is greater (see next section and Fig. 4), and their functions and associated enzymes have been characterized in much less detail [24], [50], [51]. However, pathway distributions, in particular the widespread presence of the glycine cycle, continue to suggest the reductive pathway as the archaeal ancestral form. In bacteria this cycle appears to be a good indicator for the presence of the reductive pathway, with nearly all (213 out 217) strains that have a complete glycine cycle showing either the complete (Red) or alternate (Red(-2)) forms of the direct reductive pathway. Among non-methanogenic archaea a majority has a complete or nearly complete cycle. Of these, some show a complete reductive pathway, while most lack only the genes specific to bonding at 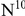 of THF (reactions 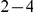 in Fig. 2). As the syntheses of archaeal (non-THF) pterins and of THF use different enzymes starting from the first commited step from GTP [50], [51], suggesting a deep evolutionary divergence of these molecules, it does not seem completely surprising that enzymes performing the equivalent pterin-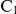 interconversions would not show up in homology searches against the THF-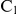 interconversion enzymes, even if this pathway is in reality present and active. This underscores the importance of further characterization of archaeal pterin-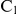 chemistry (see also Text S1).

Among archaea a complete oxidative pathway occurs only in the absence of a glycine cycle (a stricter version of a similar alternation in bacteria), and the majority of these cases are found among methanogens whose 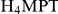-based 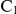 pathways play fewer biosynthetic roles than their THF homologues [24]. In the following section we combine the distribution signature with an energy analysis of the different pathways, from which it can be seen that the loss of the glycine cycle in methanogens was probably a result of pterin optimization within this derived subgroup of Euryarcheota.

### Energy optimization and oxygen toxicity

Genes for the 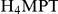-methanofuran system that interconverts oxidation states of one-carbon units have been found in both archaeal and bacterial clades, and this observation has been the basis for some hypotheses that they were present in the LUCA [52]–[54]. Several lines of evidence, however, argue against this conclusion. The most direct counterevidence comes from the synthesis and structural variation within the folate family, to which both THF and 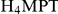 belong. Both structures are derived from GTP, but their synthetic pathways diverge as shown in the left panel of Fig. 4 [55]. Whereas in the synthesis of THF the final steps are simply the addition of an aminobenzoate and one or more glutamates, followed by the reduction of a double bond, in the case of 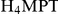 the aminobenzoate is first combined with a phosphoribosyl pyrophosphate (PRPP) before being incorporated at the homologous step [56]. This is then followed by the addition of a second PRPP, an 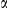-ketoglutarate [56], two methyl groups derived from SAM [57] and finally the reduction of the same double bond, leading to the same chirality [58], as in THF. The synthesis of 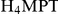 is thus an elaborated version of the synthesis of THF, which has led 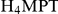 and its structural variants (shown in the right panel of Fig. 4) [59]–[64] to be termed ‘modified folates’ [65].

The variation within this group is based around two central structural features that likely affect 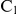 chemistry. Combining aminobenzoate with PRPP eliminates the electron-withdrawing carbonyl group of aminobenzoate (indicated in red in Fig. 4), raising the electron density at 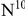 in the final pterin structure and lowering the free energies of the bound 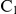 units in the direct reduction of 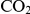 [24]. The addition of structural methyl groups (indicated in purple in Fig. 4) results in steric hindrance and a more rigid pterin structure for 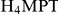 than for THF, in turn lowering the conformational entropies of pterins containing single-bound 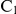 units [24]. The order of these modification in the synthesis of 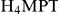 and the nature of the observed variation within the family of modified folates – elimination of the carbonyl group is universal outside of THF; the number of methyl group varies from zero to two in the same order in which they are added in 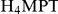 – suggests a step-wise exploration of folate modification. Further variation outside of these two aspects mostly occurs within a molecular appendage which is far removed from the active site and is thus unlikely to affect local 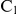 chemistry.

Within these general outlines, a wide range of structural variants is observed within this class of molecules (see Fig. 4). Of these, only THF and 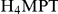 are observed within the bacteria (the latter only in a few clades), while *all* are observed within the archaea. The general premise that diversity remains highest in the domain of origin, and the fact that 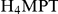 is an apparent end-point in the step-wise modification within this class of molecules suggests already that the exploration in folate modifications occurred within the archaea, and that genes for 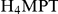 were subsequently laterally transfered to bacteria. This hypothesis is further strengthened if we consider the ecological roles of the 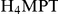 metabolic machinery, which are very different in the two domains. Methanogenic archaea use this machinery in both the oxidative and reductive direction, with autotrophs among this group using it exclusively in the fully reductive direction starting from 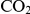, which as we previously showed was most likely the ancestral function of folate chemistry. In bacteria, by contrast, the methanopterin system is used exclusively in the oxidative direction, either as part of a methylotrophic metabolism or possibly to serve in formaldehyde detoxification. Even methylotrophic bacteria that have been classified as autotrophs first oxidize reduced forms of carbon before feeding it into traditionally recognized 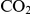-fixing pathways such as the Calvin-Benson Cycle [66]. To summarize: not only do we find a much greater structural diversity within the modified folates (including a range of apparent structural intermediates between THF and 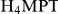) present in the archaeal domain, we also find the likely ancestral function of this whole class of molecules preserved in 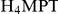 exclusively within this domain. Variations in the synthesis, structures and ecology of the folate family are all thus explained consistently by an emergence within the archaea and subsequent transfer to the bacteria, as they would not be by the reverse transfer from bacteria to archaea, or by the presence of 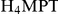 in the LUCA.

Finally we briefly discuss phylogenetic studies of the distribution of 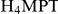 genes, some of which have been used to argue against the transfer of 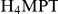 genes from archaea to bacteria. While several phylogenetic studies [53], [67] led to the assumption that the most probable scenario was a transfer from archaea to bacteria [53], [67]–[69], one study reached a different conclusion [54]. In this work, unrooted trees were built for 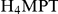 genes found within the Planctomycetes, proteobacteria, and methanogenic archaea. It was then argued that because all three clades separate in most such trees, transfer from methanogens to bacterial clades (including sequential transfers) could be eliminated, leading to the conclusion that these genes were most likely present in the LUCA [54]. We first note that this study does not address variations in the structure and synthesis of the modified folates or their ecological roles as just discussed. In addition, in any tree with just three clades, the topology automatically renders any two clades monophyletic relative to the third, which means that transfer from methanogens to the ancestor of proteobacteria and Planctomycetes cannot in fact be eliminated on these topologies alone. There are several trees in [54] that show non-methanogenic archaea branching between methanogens and bacterial clades, but these refer to genes for the biosynthesis of 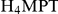, which because modified folates are common across archaea are less useful in distinguishing these scenarios. Finally, transfer from archaea to bacteria explains the pattern of absence of 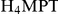 genes in most bacterial clades, which in the case of a presence in the LUCA would require an unexplained mechanism for massive differential loss within the bacteria [68]. Thus we conclude from a wide range of evidence that incorporates phylogenetics *and* the synthesis, structural diversity, and ecology of the folate family, that 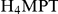 emerged within the archaea, and that this particular modified folate (but not others) was subsequently transferred to bacteria, where in a few clades it was adopted in a later derived functional role.

If the folate family diversified within the archaea as we have proposed, through step-wise modification to the synthesis of THF, what then drove these modifications? As alluded to in the description of the individual key structural changes, we argue here that it was an energetic optimization of the fixation of 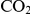 through direct reduction. We calculated the biosynthetic cost of glycine and serine in units of ATP and reductant (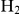 equivalents) for both the reductive and oxidative pathways, shown in Table 2. Since the cost of the oxidative pathway rises with general cost of fixing carbon for different primary fixation pathways, while the reductive pathway has a fixed cost, Table 2 only shows cost for the oxidative pathway as part of the two most energy efficient autotrophic strategies, the rTCA cycle and the reductive acetyl-CoA pathways [1], [70]. It can be seen that the only autotrophic context in which the oxidative pathway is more efficient than the reductive pathway is methanogenesis. The combined effect of higher electron density on 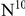 of 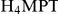 attained by fusing aminobenzoate with PRPP before incorporation, the lowering of the entropy of single-bound 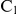-folate structures through the addition of structural methyl groups – both of which may have on average conveyed an energetic advantage in isolation – and the usage of the methanofuran thus resulted in a reduction of the number of ATP's required for the uptake of 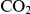 through this pathway. The higher electron density at 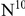 of 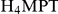 that facilitates the uptake of 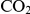 without ATP hydrolysis increased the stability of the C-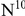 bond, but thereby sacrificed the capacity to donate the methylene group and synthesize glycine directly, explaining the absence of the glycine cycle in methanogens. (We have noted that, in contrast, most non-methanogens show all genes for this cycle.) In addition, the lowering of the entropies of single-bound 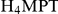-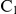 molecules may contribute to the smaller free energy differences between these and closed ring (methenyl- and methylene-) 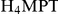-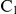 structures, allowing easier transitions between 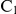 oxidation states and thus facilitating reversal of pterin chemistry from the reductive to the oxidative direction [24]. This robust change in functionality would also explain why only 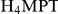 among all the modified folates was transfered from archaea to bacteria. Finally, as we shall see below, energy optimization through elimination of redundant ATP-consuming reactions was likely a more general selective force that also explains the initial emergence of WL phenotypes.

**Table 2:** ATP and reductant cost for synthetic routes to glycine and serine.

	Glycine	Serine
Red	1 ATP+3 Red	2 ATP+5 Red
Ox, in:		
rTCA	3 ATP+4 Red	3 ATP+5 Red
WL 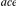	2 ATP+4 Red	2 ATP+5 Red
WL 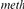	1 ATP+4 Red	1 ATP+5 Red

We interpret the pathway distributions as showing, generally, that energy optimization is a secondary selection force to oxygen toxicity, when the latter is present. For example, once acetyl-CoA synthase was eliminated in the direct reduction of 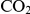 upon oxygen exposure in deep-branching autotrophs, modification within the folate family as just described would no longer have been advantageous as the oxidative synthesis of glycine and serine could no longer be connected to folate-mediated direct reduction of 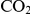, eliminating this route to lowering ATP cost. Another example is the cyanobacteria, which use the glyoxylate pathways to synthesize glycine and serine even though energy accounting suggests that a hybrid strategy involving the reductive pathway would be more economical than using glyoxylate emerging out of the CBB cycle, which has one of the highest ATP costs of carbon-fixation [1], [70]. However, it is known that 2-phosphoglycolate, the precursor to glyoxylate in these organisms, is formed when 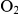 replaces 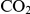 in the CBB Rubisco reaction, and subsequently inhibits the cycle [30]. In this case the adoption of the glyoxylate pathway thus furnishes a mechanism to remove 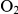-induced growth inhibition and to recycle 2-phosphoglycolate through anabolism. We predict that the reductive pathway retains a role in cyanobacteria living under anoxic [71] or high-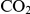 [30] conditions, where the CBB cycle does not produce (significant) 2-phosphoglycolate, or in mutants where the glyoxylate pathways have been deactivated [30].

### A complete reconstruction of the evolutionary history of carbon-fixation

Having established that direct reduction of 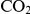 is the most likely ancestral metabolic pathway to glycine and serine, as well as the ancestral function of THF-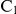 chemistry in general, we next consider a full evolutionary reconstruction of carbon-fixation. We first note, as briefly mentioned in the introduction, that with these new results the deep-branching rTCA and Wood-Ljungdahl carbon-fixation phenotypes show a high degree of similarity. For deep-branching rTCA phenotypes we find parallel use of an incomplete WL pathway that lacks only the final synthesis of acetyl-CoA, which as noted previously is catalyzed by one of the most oxygen sensitive enzymes in the biosphere. Anaerobic WL phenotypes, in contrast, do possess this complete sequence to acetyl-CoA, and they then complete the set of universal anabolic precursors through a variety of incomplete rTCA cycles. Closer inspection of these incomplete cycles shows that while connection to the universal anabolic precursors is always maintained, in all cases the usage of one of the redundant ATP-dependent steps involved in thioester bond formation is eliminated: the synthesis of citryl-CoA from citrate (which is subsequently cleaved to acetyl-CoA and oxaloacetate), or the synthesis of succinyl-CoA from succinate [72], [73]. The incomplete WL pathway as part of the hybridized deep-branching rTCA cycle is thus associated with oxygen sensitivity, while the incomplete rTCA cycles as part of deep-branching WL are associated with ATP economy. The template that underlies both is a fully connected rTCA-WL network.

The modular role and ancestral status of direct 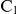 reduction thus anchors the most fundamental division in carbon fixation strategies, between the autocatalytic rTCA loop and the non-autocatalytic reductive acetyl-CoA pathways, and suggests that the linked rTCA-WL network preceded both. From the linked rTCA-WL phenotype, oxygen toxicity to the acetyl-CoA synthase causes divergence of *Aquifex*-like rTCA phenotypes, while energy optimization through elimination of redundant ATP-dependent citryl-CoA or succinyl-CoA synthetases leads to the divergence of WL phenotypes. Other fixation pathways may be derived from these by loss or addition of only a few key reactions, linked again to ATP, oxygen sensitivity, or in some cases alkalinity or redox states. Within our basic assumption that the biosphere has always been autotrophic from 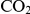, we may therefore ask: 1) whether all carbon-fixation phenotypes may be connected while maintaining uninterrupted access to all initial anabolic branching points from 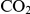; 2) whether these connections can be made with no repeated innovations; 3) which networks that are unobserved in extant biology must be posited to connect all networks that are observed. These questions are answered by arranging the known phenotypes, and the new hybrid forms revealed here, as nodes on a tree according to parsimony, as outlined in Fig. 1D, where links represent evolutionary innovations.

**Figure 5:**
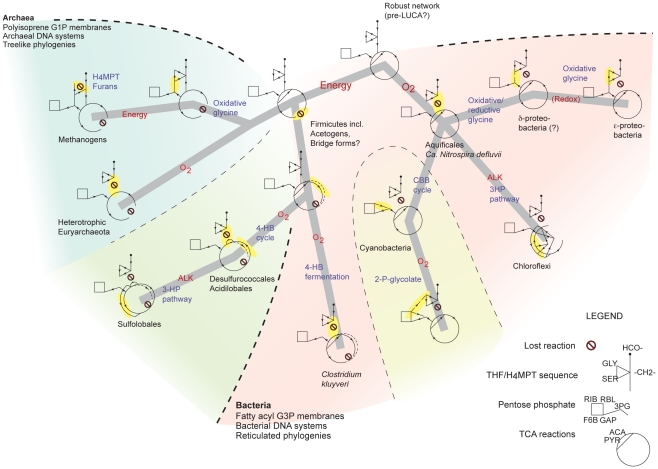
Phylometabolic tree of carbon-fixation. The grey line represents the maximum-parsimony tree linking the major metabolic phenotypes (diagrams). Three major modules shown schematically (see the legend) are pterin/folate-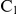 metabolism, the pentose-phosphate pathway, and the TCA cycle reactions. Lost reactions (symbol) include the acetyl-CoA synthase (in 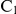 metabolism), and ferredoxin-dependent succinyl-CoA synthase (in TCA loop) or citryl-CoA synthase (not shown). Abbreviations: 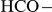, formyl; 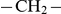, methylene; ACA, acetyl-CoA; PYR, pyruvate; GAP, glyceraldehyde-3-phosphate; F6B, fructose-1,6-bisphosphate; RIB, ribose-phosphate; RBL, ribulose-phosphate; ALK, alkalinity; others as in Fig. 2. Arrows indicate reaction directions; dashed line connecting 3PG to SER indicates intermittent or bidirectional reaction. Highlighted in yellow are the innovations underlying divergences, while red labels on links indicate evolutionary force associated with each innovation, explained in the text. Beneath each diagram is an extant species or clade name where the phenotype is found. Colored regions indicate domains within which all known instances of the indicated phenotypes are restricted.

A maximum-parsimony tree connecting all known autotrophic pathways, in which all nodes represent viable carbon-fixation phenotypes, is shown in Fig. 5. We note first the position of the linked rTCA-WL network between rTCA and WL phenotypes. Fully connecting the deep tree of life while maintaining 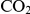 autotrophy requires connecting rTCA and WL phenotypes. No single change can connect them while maintaining uninterrupted access to all essential branching points from 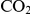, and the only sequence of two changes that does maintain autotrophy passes through the linked rTCA-WL network. The ancestral state required to connect the network is thus consistent with the alternative selective filters from oxygen and ATP use discussed above. From this inserted node, we may then connect all other observed carbon-fixation phenotypes, with no further insertions needed, through sequences of single changes for which we can invoke plausible ecological causes.

If we assume that once the complex, ATP-dependent, citryl-CoA or succinyl-CoA synthetase enzymes were lost they could not easily be recovered, we can then explain the absence of the rTCA cycle in the archaea, together with the curious, chimeric Dicarboxylic/4-Hydroxybutyrate (DC-4HB) and 3-Hydroxypropionate/4-Hydroxybutyrate (3HP-4HB) cycles as the only autocatalytic loop pathways in this domain [1], [2]. Once autocatalytic rTCA cycling has been lost (as in the emergence of WL phenotypes), organisms can only survive subsequent loss of the acetyl-CoA synthase due to oxygen exposure if they either can survive as heterotrophs, or else possess a latent cycle that can be activated. Indeed, in the Euryarcheota all non-methanogens are heterotrophs lacking acetyl-CoA synthase. In the Crenarcheota, in turn, the DC-4HB cycle shares the first arc of the rTCA cycle (acetyl-CoA
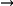
succinyl-CoA) but not the second (succinyl-CoA
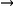
acetyl-CoA), and it has a significantly higher ATP cost per carbon fixed than does rTCA [1], [70]. Therefore, an ancestral status for DC-4HB cannot be motivated as requiring fewer chemical innovations, nor could this pathway have competed with rTCA energetically. If, however, a subgroup of WL organisms that possessed all the steps of the second 4HB arc for other purposes were exposed to oxygen, the activation of the cycle and the transition to this carbon-fixation phenotype would be enforced to maintain autotrophy from 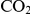. The second arc of DC-4HB originates from acetate along the isoprene synthesis pathway essential to archaeal lipids, and from succinate along a fermentative pathway using the key 4-hydroxybutyryl-CoA dehydratase [74]–[76]. Both of these sequences have been found in the Clostridial clade of the Firmicutes [75], [76], which also contains many WL organisms, supporting this scenario. The most elaborate case is that of *Clostridium kluyveri*, which contains pathway segments to reach all DC-4HB intermediates, though these are not used as part of an autocatalytic cycle, instead supporting a fermentative heterotrophic metabolism [76].

In a similar fashion, the 3HP-4HB phenotype then branches from the DC-4HB phenotype through replacement of the first rTCA arc (acetyl-CoA
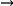
succinyl-CoA), which was still preserved in DC-4HB. The central difference, from the perspective of carbon-fixation, between the rTCA arc and the 3HP arc that replaces it is that the latter requires only uptake of bicarbonate whereas the former takes in 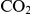 as well. The transition to 3HP-4HB thus appears to be driven by changes in the alkalinity in the environment, with an equilibrium shift from dissolved 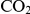 toward bicarbonate favoring the emergence of the 3HP pathway. The subsitution of the second rTCA arc by the 4HB arc within the crenarcheota had already removed all reactions involving 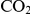 in the second half of the pathway, thus imposing no further barriers to the transition from DC-4HB to 3HP-4HB in response to alkalinity. The archaeal case contrasts with the emergence of the 3HP pathway in bacteria, as part of the 3-HP bi-cycle, which similarly uses only bicarbonate uptake and has thus also been recognized as an adaptation to alkaline conditions. In the latter case, both (rTCA) arcs in the initial phenotype utilized 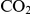 uptake reaction, and adaptations to avoid these resulted in a more complex pathway structure. In addition to the appearance of the 3HP arc this involves the reversal of part of the first rTCA arc and the appearance of an alternate completion of the 3HP arc through combining with a glyoxylate that is one of the products of the reversed (first) rTCA arc. We note, however, that these complex pathways result from the relatively simple and uniform chemistry of aldol reactions, and that part of the 3HP-bicycle overlaps with the glyoxylate shunt, which is a similar bicycle.

The emergence of the 3HP pathway in both archaea and bacteria as just discussed is one of the two main parsimony violations in Fig. 5. The 3HP pathway uses two key biotin-dependent carboxylation reactions (to malonyl-CoA and methylmalonyl-CoA), which suggests a bacterial origin as this enzyme class features prominently in the biosynthesis of the fatty acids that make up bacterial membranes, in contrast to archaeal lipids, which are based on isoprenoid backbones [77]. However, a comparison of enzymes for thioesterification of propionate to propionyl-CoA has been interpreted as implying convergent evolution for these essential steps in the pathway [78]. Since the appearance of the 3HP pathway is recognized as an adaptation to alkaline conditions in both bacteria and archaea, either convergence or LGT is plausible due to restricted common environments. The other main parsimony violation in Fig. 5 is the parallel emergence of the oxidative route to serine. Oxidative synthesis of serine involves three of the most ubiquitous reaction types/enzymes within metabolism, the dehydrogenation of an alcohol to a carbonyl (reaction 11), a transamination of that carbonyl to an amine group (reaction 10), and a dephosphorylation (reaction 9). The recurrent emergence of this pathway through either duplication or promiscuity of common enzymes is therefore not likely to be a low-probability event, and thus not a significant violation of the assumptions behind a maximum-parsimony reconstruction.

The tree of Fig. 5 separates the Firmicutes together with all archaea as the branch from the linked rTCA-WL network originally driven by energy optimization in the absence of oxygen. All other bacterial lineages separate by an early loss of acetyl-CoA synthase. This basic division – with Firmicutes separate from all other bacterial clades – is supported by phylogenetic studies that focus on directed insertion-deletions in paralogous informational and operational genes [79], and on universal orthologous genes [41]. The fact that all archaea separate as a monophyletic branch from a pool of ancestral bacteria may be associated with the more tree-like archaeal phylogeny, compared to the more web-like phylogeny of bacteria, which suggests higher rates of LGT in the latter [21]. The co-evolution of the archaeal DNA-replication system together with less permeable isoprenoid membranes [77], [80], resulting in lower LGT in this domain, are possible mechanisms for isolation.

### The ancestral carbon-fixation phenotype

The carbon-fixation phenotypes at all internal nodes of our reconstructed phylometabolic tree are found preserved in extant organisms, except for the linked rTCA-WL network at the root node. While many of these derived nodes incorporate parallel fixation pathways, in all such cases the parallel pathways are disjoint and supply carbon to distinct portions of biomass. The linked rTCA-WL network is qualitatively different, having two separate input channels that supply acetyl-CoA, resulting in a kind of metabolic redundancy. We must ask, does our choice to insert an unobserved node, in order to connect the tree while preserving autotrophy, justify the reconstruction of an ancestor with a form of redundancy not found in any of its descendants, and if so, what does this reconstruction tell us about the evolution of earliest life? We address both questions by considering the relation of network topology and redundancy to self-amplification.

The capacity of life for exponential growth, resulting from proportional self-amplification by metabolic and other networks with “autocatalytic” topology [81], [82], is essential to self-repair and robustness in the face of perturbations. At the small-molecule substrate level the rTCA cycle is network-autocatalytic, and thus capable of exponential growth above a threshold rate of production of acetyl-CoA, but also fragile against collapse if production falls below this threshold [83]. The threshold fragility, which may have been a more serious problem in an era of primitive catalysts or regulation, is removed while preserving autocatalysis if acetyl-CoA is independently supplied by WL. Conversely, WL in modern organisms may be considered network autocatalytic at the level of whole-cell physiology including enzymes and cofactors, but it relies on the integrity of synthetic pathways for these molecules which are more complex even than the small-molecule substrate. rTCA, which provides an independent channel for carbon fixation and synthesis of precursors, would thus reciprocally support the robustness of WL in the earliest era of organic (versus proposed earlier mineral [69], [84]) cofactors.

The difference in network topology between our reconstructed root, and its derived descendants, would then reflect a shift in the character of natural selection acting on the earliest versus later cells. In early cells (or pre-cells) with imprecise or unreliable enzyme function, consequent leaky pathways and fluctuating cofactor concentrations, or unreliable regulation of anabolism, robustness inherent in the topology of the substrate network would have carried a selective advantage. (Note that anabolism is a form of “parasitic side-reaction” from rTCA cycling, and that an inadequately regulated anabolism can as readily carry carbon fixation below the threshold for self-maintenance, as external factors can). As each of these cellular-level mechanisms was refined, redundant self-amplification of small-molecule substrates would have become unnecessary, and conservation of ATP or adaptation to oxidizing environments would have become more advantageous.

These observations also motivate our reference to the linked rTCA-WL network as a “root” phenotype in a maximum-parsimony tree that, by its construction method, is otherwise unrooted. All other phenotypes in the tree may be explained as evolutionary diversifications *away* from the linked rTCA-WL network, which is both a template for these divergences, and because of its redundancy, a more plausible candidate for a primitive ancestral form than any modern phenotype. The good overlap of our carbon-fixation tree with the later branchings of bacteria and archaea indicates the fundamental role of autotrophy in shaping the deep evolution of the biosphere, and suggests that the later nodes describe not only cellular life, but emerging well-resolved clades. The less-clear separation of bacteria and archaea near the root in Fig. 5, the correspondence of these branches to the reticulated domain of gene phylogenies [12], [14], [19], the need and character of the inserted root node, and the flexible interpretation of our carbon-fixation phenotypes as species or consortia, leave open the possibility that the earliest branches were stages of chemical evolution that preceded modern life [3]–[6].

### Parsimony, the role of ecosystems, and the rise of oxygen

Our phylometabolic reconstruction, and the surprising tree that it yields, has focused on the particular function of carbon fixation. However, the same methods could be extended to a fuller description of core metabolism, and from the phenotypes we have already shown, we may anticipate certain specific complications that will be introduced with a wider reconstruction. These reflect the changing nature of ecological interactions with increasing oxygenation, and they give added insight into the interpretation of the high degree of parsimony we have shown for innovations in carbon-fixation, most of which took place in anaerobic or micro-aerobic conditions.

For any phylogenetic reconstruction, it is important to remember that the nodes and links on a tree are *summary statistics* for relatedness of samples taken from a population process that may have been very complex. A high degree of parsimony in a tree does not indicate the absence of complex structure in populations, constraints on innovation, or ecological interactions; at most it indicates a lack of specific evidence that innovation required anything more than rare variations and environmental selection in vertically transmitted phenotypes. The cases in which violations of parsimony are inescapable provide evidence that multiple levels of organization were causally essential to the course of innovation, whether these were latent constraints causing some innovations to recur (leading to evolutionary convergence), or ecological interactions leading to gene or pathway transfer. The structure of parsimony violation then indicates what forms of multilevel interaction must be deduced to explain evolutionary causation.

Two illustrative cases of parsimony violation that we have elaborated are the transfer (or combined transfer/convergence) of the 3HP pathway, and the gradual elaboration of pterin cofactors followed (as we argue) by the late transfer specifically of 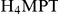 from archaea to bacteria. The parallel innovation of the 3HP pathway in *Chloroflexi* and in the Chrenarchaeota entails duplication of an entire (and rather elaborate) pathway segment, and not merely a single key gene. It is favored in specialized environments which we would expect to create long-term association between inhabiting species, these environments contain a stressor (alkalinity) which we expect to induce gene transfer, and relative to the very ancient divergences in our tree, the innovations of 3HP occur late, at an era when we expect organism lineages to have evolved refractoriness to many forms of gene transfer [85]–[87], along with more integrated control of chromosomes. Similar long-term associations in anaerobic environments such as coastal muds are believed to have led to the (otherwise uncommon) aggregate transfer of a large complement of operational genes from 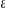-proteobacteria to Aquificales [88].

The transfer of 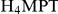 reflects an even more fundamental link between oxygen and ecosystem structure. As it occurs in methanotrophs and methylotrophs, 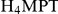 is used for oxidation of methane and other reduced 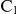 species, the most extensive form of heterotrophy of reduced carbon, which relies on environmental oxidants to link methane producers and consumers. This function, driven by trophic interactions, is layered over the foundation of 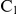 reduction on folates which we reconstruct as ancestral in the clades harboring methanotrophs and methylotrophs. The complexity of methylotrophy [89] anticipates the enormous diversification of catabolic pathways that becomes available with oxidation of reduced biotic carbon [17], but which depends on details of ecological provision and accessibility of carbon sources.

Finally, it is interesting that many of these complexities would fall cladistically near the tips of the tree in Fig. 5, suggesting that innovation in carbon fixation ceased, to be replaced by innovations in carbon exchange through ecosystems, on a horizon coinciding with the rise of oxygen. This pattern brings into sharper relief the striking lack of multilevel dynamics that would distinguish organism and ecosystem roles in the reconstruction we have shown.

### Phylometabolic analysis: future directions

In this paper we have demonstrated a novel method that integrates constraints from FBA and from phylogenetics. Individually, metabolic and phylogenetic reconstructions are both subject to ambiguities, especially for deep-branching lineages with significant gene divergence from well-characterized model organisms, and extensive LGT near the root [12], [14], [19]. Integrating the two can resolve ambiguities inherent in each, as well as providing new ways to tailor questions to specific features of early evolution.

Here, as a proof of principle, we have presented a coarse-grained reconstruction of the input channels of carbon into the biosphere only up to the initial anabolic branching points. We can relate all modern forms of carbon-fixation to a single ancestral form, and we find that innovations in carbon-fixation were the foundation for most major early divergences in the tree of life. We have also proposed specific causes for the major divergences, and argued for a very small role for lateral gene transfer or convergent evolution. The absence of any phylogenetic signature of important ecological co-evolution for this function, combined with selective forces that originate in energetics or inorganic chemistry, offers additional specific links between genome evolution and the geological record [15]. The consistency of the reconstruction demonstrates, with specific examples, how the fine structure of organic chemistry and geochemistry can enter as detailed constraints on long-range evolutionary dynamics. The hypotheses required by the reconstruction further imply several sets of specific experimental predictions, which are outlined in the Text S1.

By limiting our reconstruction to the networks of core carbon fixation – and by virtue of the modular interface of this function with later stages of biosynthesis – we have selected a problem for which many distinctions between organism and ecosystem (any that do not leave phylogenetic signatures) may be passed over, which falls prior to most ambiguities from reticulated gene phylogenies, and which leads to a high-parsimony tree that can be identified manually. The extension of these methods to larger networks with more ambiguity, and to historical reconstructions for which trees provide less adequate representations of complex population processes, will require formal probability models, which may be implemented in joint-maximum-likelihood or Bayesian MCMC phylometabolic algorithms. The current work suggests ways to increase the number of dimensions of “meaning” that can be used to define such probabilistic models, by placing genes in both physiological and ecological context. In this way we may reconstruct trees of life that reflect more of the multi-level character of evolution and development than is suggested by gene counts, and that capture constraints which may have acted continuously since its emergence.

## Methods

For a description of the basic principles of phylometabolic analysis, see the introduction and Fig. 1. This method rests in part on metabolic flux-balance analysis (FBA), which has been described in detail elsewhere [90], [91]. Briefly, the core of FBA consists of the following three equations:
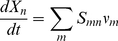
(1)

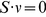
(2)

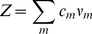
(3)where 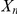 is the concentration of metabolite *n*, 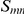 is the stoichiometry of metabolite *n* in reaction *m*, and 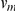 is the flux of that reaction. The *n* 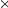 *m* matrix *S* and the *m*-dimensional vector 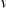 are the the stoichiometries and fluxes of the total metabolic network. Under steady state growth, the principle of mass balancing can then be expressed as equation (2). Finally, *Z* is an objective function that is selected for optimization, and consists of a linear combination of individual individual fluxes weighted by proportionality constants 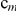. Z is often chosen to be the biomass composition of an organism, and maximizing its output thus maximizes growth. The full metabolic network of an organism is reconstructed from its annotated genome. While optimization and matching to laboratory growth data of specific organisms requires detailed analysis of the constraints of individual reactions and careful computational modeling, the initial reconstruction of the network requires only that the network represents a viable metabolism capable of supporting growth (*Z* 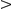
0).

Our analysis is restricted to the access (from 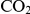) of the universal anabolic precursors that represent the initial branching points in anabolism, requiring as in the initial reconstruction of an organism only that they are produced (*Z* 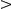
0). These anabolic branching points are acetyl-CoA, pyruvate, oxaloacetate, succinyl-CoA and 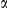-ketoglutarate, which is a very small number of intermediates to consider, and because studies of carbon-fixation phenotypes have already shown that they are in all cases reached through reuse of partial TCA sequences in fact no computational analysis is required. As explained in the main text, the only intermediates accessible through pathways that circumvent all the universal anabolic precursors are glycine and serine, so our analysis of metabolic genes focusses on the pathways to these intermediates. Three main pathways to glycine and serine are known (see Fig. 2 and the Results and Discussions section), and we analyze the gene profiles for a large number of species for the presence of the necessary enzymes for each of these three pathways.

The gene profiles for all strains used in this study were obtained from the Uniprot database [35]. For all complete annotated genomes within each clade, gene profiles in the three pathways were obtained by searching for enzyme classes (through EC numbers) and names. The searches were done in a redundant manner to ensure that all naming variations in the annotation of a given gene/enzyme were included in the final result. While in general we used the annotations as given in the database, in a few cases a (Uniprot built-in) BLASTp search was done to confirm the absence or presence of an enzyme if the profile of a particular strain seemed to contradict the pattern of the clade overall. These are highlighted in Table 1 and Table S1 in Text S1. If within a particular strain all enzymes of a given pathway showed up in our database search the pathway (either active or latent) was counted as present in that strain.

The EC numbers and one common variation of the full names of the numbered reactions as shown in the various Figures, and throughout the text are as follows: 1 = formate dehydrogenase (EC: 1.2.1.2); 2 = 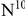-formyl-THF synthase (EC: 6.3.4.3); 2A = 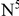-formyl-THF cycloligase (EC: 6.3.3.2); 3 = methenyl-THF cyclohydrolase (EC: 3.5.4.9); 4 = methylene-THF dehydrogenase (EC: 1.5.1.5); 5 = dihydrolipoamide dehydrogenase (EC: 1.8.1.4); 6 = aminomethyltransferase (EC: 2.1.2.10); 7 = glycine dehydrogenase (EC:1.4.4.2); 8 = serine hydroxymethyltransferase (EC: 2.1.2.1); 9 = phosposerine phosphotase (EC: 3.1.3.3); 10 = phosphoserine aminotransferase (EC: 2.6.1.52); 11 = 3-phospho-glycerate dehydrogenase (EC: 1.1.1.95); 12 = alanine-glyoxylate transaminase (EC: 2.6.1.44).

## Supporting Information

Text S1We present several sets of experimental predictions of our work, and a more detailed version of Figure 2. In addition, we present tables containing examples of gene-profiles in the glycine/serine synthesis pathways for several characteristic deep-branching organisms, and distributional evidence for a suspected alternate route for formate uptake through 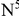 rather than 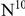 of THF.(PDF)
