# Supplementary material for: The Emergence and Early Evolution of Biological Carbon-Fixation
Source: PLoS Comput Biol. 2012 Apr 19;8(4):e1002455. doi: 10.1371/journal.pcbi.1002455 (PMC3334880; doi:10.1371/journal.pcbi.1002455)
Supplement: Text S1 — We present several sets of experimental predictions of our work, and a more detailed version of Figure 2. In addition, we present tables containing examples of gene-profiles in the glycine/serine synthesis pathways for several characteristic deep-branching organisms, and distributional evidence for a suspected alternate route for formate uptake through rather than of THF. (PDF) [file pcbi.1002455.s001.pdf]

Text S1

Supporting Methods

The full names of the organisms as listed in Table S1 and mentioned in the main text are as follows (with three letter abbreviations in parentheses):

Bacteria - Aquificales: *Hydrogenobacter thermophilus* (HTH), *Thermocrinis albus* (TAL) and *Aquifex aeolicus* (AAE), Thermotogales: *Fervidobacterium nodosum* (FNO), *Thermotoga maritima* (TMA) and *Thermotoga neapolitana* (TNE), Firmicutes: *Thermoanaerobacter tengcongensis* (TTE) and *Moorella thermoacetica* (MTA), Chloroflexi: *Chloroflexus auriantiacus* (CAU) and *Chloroflexus aggregans* (CAG), Nitrospirae: "Candidatus *Nitrospira defluvii*" (NDE) and *Thermodesulfovibrio yellowstonii* (TYE), Chlorobia: *Chlorobium chlorochromatii* (CCH) and *Chlorobium phaeobacteroides* DSM 266 (CPH), Planctomycetes: *Isosphaerae pallida* (IPA), Cyanobacteria: *Prochlorococcus marinus* (PMA) and *Nostoc punctiforme* (NPU).

Crenarcheota - Thermoproteales: *Thermoproteus neutrophilus* (TNE) and *Pyrobaculum arsenaticum* (PAS), Desulfurococcales: *Thermosphaera aggregans* (TAG) and *Hyperthermus butylicus* (HBE), Sulfolobales: *Sulfulobus solfataricus* (SSO) and *Metallosphaera sedula* (MSE). Korarcheota - "Candidatus *Korarchaeum cryptofilum*" (KCR). Euryarcheota - Methanogens: *Methanocaldococcus jannaschii* (MJA), *Methanosarcina barkeri* (MBA) and *Methanospirillum hungatei* (MHU), non-Methanogens: *Haloarcula marismortui* (HMA), *Halogeometricum borinquense* (HBO), *Thermoplasma acidophilum* (TAC) and *Pyrococcus abyssi* (PAB).

Supporting Discussion

The phylometabolic analysis described in the main text suggests several important experiments. We outline four main sets here.

Confirmation of two alternative C_1 reductive pathways in deep-branching, non-WL, organisms

The first class of experiments concerns confirming the operation of the direct reductive pathway outside of WL organisms. We highlight several non-WL organism here and in the main text with a complete gene complement in the reductive pathway that can be examined for this purpose, including members of the Thermotogae, Nitrospirae, and Chloroflexi. In particular, however, we focus discussion here on elucidating the mechanisms involved in the alternate form of the reductive pathway that we find in a large number of strains. In this alternate form the gene for reaction 2, the ATP-consuming synthesis of N^{10}-formyl-THF (see Fig. S1), is the lone missing gene in an otherwise complete pathway. Remarkably, nearly a quarter of all bacterial strains (and in many clades a much higher fraction still) show the presence of this version of the reductive pathway. As shown in Supplementary Table 2, we find that a large majority of these strains instead possesses a gene for N^5-formyl-THF cycloligase, an enzyme whose origin and metabolic role remains very poorly understood. We also note the very broad distribution of this gene across all deep bacterial and archaeal clades.

Based on these observations we propose a possible alternate route to formate incorporation and reduction on THF – through N^5 rather than through N^{10} (see Fig. S1) – in deep-branching bacteria and archaea. This route would involve either a previously unidentified N^5-formyl-THF synthase, or broader functionality of the N^5-formyl-THF cycloligase than previously annotated (each shown as dashed lines in Fig. S1). The use of an ATP in the synthesis of N^{10}-formyl-THF is necessary due to the low electron density and low pKa of the N^{10} in folates. The less stable nature of the resulting C-N^{10} bond likely

explains the higher biosynthetic capacity of THF relative to H_4MPT, where a higher pKa of the N^{10} stabilizes the C-N^{10} bond and (together with the action of the methanofuran cofactor) removes the need for ATP hydrolysis in its formation. Similarly, N^5-formyl-THF cycloligase also requires an ATP in creating the C-N^{10} in the cyclization step. The proposed alternate route would thus conserve the total consumption of 1 ATP in the synthesis of (N^5–N^{10})-methylene-THF from free formate and THF, and would only alter the order of the C-N bond formations.

Organisms in several clades could be studied for this alternate route, including members of the Aquificales (the Aquificaea family), the Chlorobiales or the Cyanobacteria. If an N^5 incorporation route is active in these or any other deep clades, it may reflect and intermediary form between the N^{10} incorporation route in bacteria and the N^5 incorporation route of methanogens. Experiments to elucidate this chemistry could thus lead to insights into the evolutionary diversification in this important direct incorporation pathway of cellular carbon.

Phenotype switching and evolution of C_1 metabolism in Cyanobacteria

A related class of experiments is one that examines the possibility of phenotype switching in the Cyanobacteria. It is known that Cyanobacteria synthesize glycine through the glyoxylate pathways (reaction 12 in Fig. S1), but growth is nonetheless observed in mutants where the formation of glyoxylate is inhibited. More generally, it is also known that certain cyanobacterial strains can live under anoxic conditions, where 2-phosphoglycolate (the precursor to glyoxylate) is not formed. Furthermore, as outlined above, a majority of cyanobacterial strains we examined have gene profiles similar to Aquificales and Chlorobiales in the reductive pathway to glycine and serine, missing only the gene for reaction 2 but having a gene for reaction 2A.

This suggest that under anoxic conditions, or in mutation experiments, cyanobacteria may switch from the synthesis of glycine and serine in the glyoxylate pathways to the reductive pathway. This phenotype switch would in essence represent reverting from the modern autotrophic form in which all carbon is fixed through the Calvin-Benson-Bassham (CBB) cycle to the hybrid ancestral cyanobacterial form where a small fraction of carbon is fixed through the reductive pathway in parallel to the CBB cycle.

Diversification in Archaeal pterin-C_1 chemistry

A third class of experiments involves elucidating in greater detail the pterin-C_1 chemistry in archaea. Many archaeal genomes lack only the THF-interconversion genes associated with the direct reductive pathway, and, in addition, the diversity of pterins in archaea is greater than in bacteria. Variations arise both in the arylamine-appendage attached at the N^{10} position, and in the presence of methyl-groups at either of the R_2 positions as seen in Fig. S1. The absence of a carbonyl group immediately adjacent to the arylamine group in H_4MPT raises the electron density and pKa of the N^{10}-nitrogen relative to THF, and this has been suggested to be the central factor in eliminating the use of ATP in the incorporation of formate and the lower biosynthetic capacity of H_4MPT in methanogens. However, it has also been noted that steric hindrance between the two methyl-groups on H_4MPT results in a reduction of the entropic effects in reactions involving this pterin, which may also explain some part of the difference relative to THF-chemistry. We find a compete absence of the glycine cycle (reactions $5-8$) only in methanogenic archaeal clades, with an otherwise broad presence across clades that possess pterins that share some, but not all, of the substitution characteristics of H_4MPT. In addition, as mentioned above, we find a high abundance of N^5-formyl-THF cycloligase across the archaeal domain, in many cases where all other THF interconversion enzymes are absent.

Characterizing archaeal pterin chemistry in greater detail is thus in several ways important for better understanding the evolution of carbon-fixation within the Archaea. First, it would confirm the use (or non-use) of the direct reductive pathway to glycine and serine, and thus the ancestral status of this route, in this domain. In addition, systematically examining variations in the arylamine-appendage and

the substitution of methyl-groups in archaeal pterins and how it relates to the synthesis of glycine and serine would help in characterizing the relative contributions of entropic and electron-withdrawal effects in the biosynthetic capacity of the pterin group. This could in turn allow a better understanding of the greater diversity of pterin chemistry in the archaea relative to bacteria, as well as of the emergence of methanogens within the euryarcheota (as discussed in the main text).

Amino acid-specific isotope ratios and the decomposition of isotope effects in C_1 reduction

A fourth, and last, class of experiments regards using the direct reductive synthesis of glycine and serine as a means to understand variations in the isotopic shifts that are observed in different autotrophic pathways. The WL pathway is commonly cited as producing the greatest depletion in ^{13}C of all autotrophic pathways, with a depletion of order -40 permil relative to input CO_2. Since the full WL pathway comprises 9 reactions, it is of interest to disaggregate this isotope shift, to assign contributions to specific reactions performed on pterin cofactors and to the acetyl-CoA (ACA) synthase reaction at the culmination of the pathway. Sensitivity to substrate mass may contribute to more precise characterization of the reaction mechanism in the Ni-substituted Fe-S cluster of the ACA synthase including the interaction with its associated Cobalt-tetrapyrrol cofactor, or may highlight the tuning of nitrogen-bonding properties on pterins. Since direct C_1 reduction has been proposed to have mineral origins, knowledge of either type may provide clues to the transfer of reduction steps from mineral substrates to pterin/folate cofactors, and transfer of catalysis from mineral or chelated metal centers to an organometallic enzyme.

The broad and diversified role we have asserted for direct C_1 reduction among deep-branching organisms, and particularly the occurance of reduction on folates with or without the ACA synthase reaction, provide a low-cost comparative analysis to partially separate isotope effects. Identification of amino acid-specific isotope ratios would also further help refine the use of biomass compositions in characterizing newly observed species in terms of the carbon-fixation strategies used.

Any isotope shift that accumulates in the direct C_1 reduction sequence prior to the transfer of methylene will be restricted to the $\alpha-$carbon of glycine and the $\alpha-$ and $\beta-$carbons of serine, and to the serine-derived carbon of cysteine and tryptophane in non-WL organisms that use the full reductive pathway to fix part of their carbon. These isotope shifts will be present in *all* fixed carbon of WL organisms. Isotope shifts specific to ACA synthase will be present in all carbon *except* the few positions in glycine, serine, cystein and tryptophane just noted, in acetogens and other autotrophic WL organisms that use the direct reductive route to glycine and serine. The same ACA synthase isotope shift will be present in *all* carbon in methanogens and other autotrophic WL organisms that use the oxidative pathway to glycine and serine.

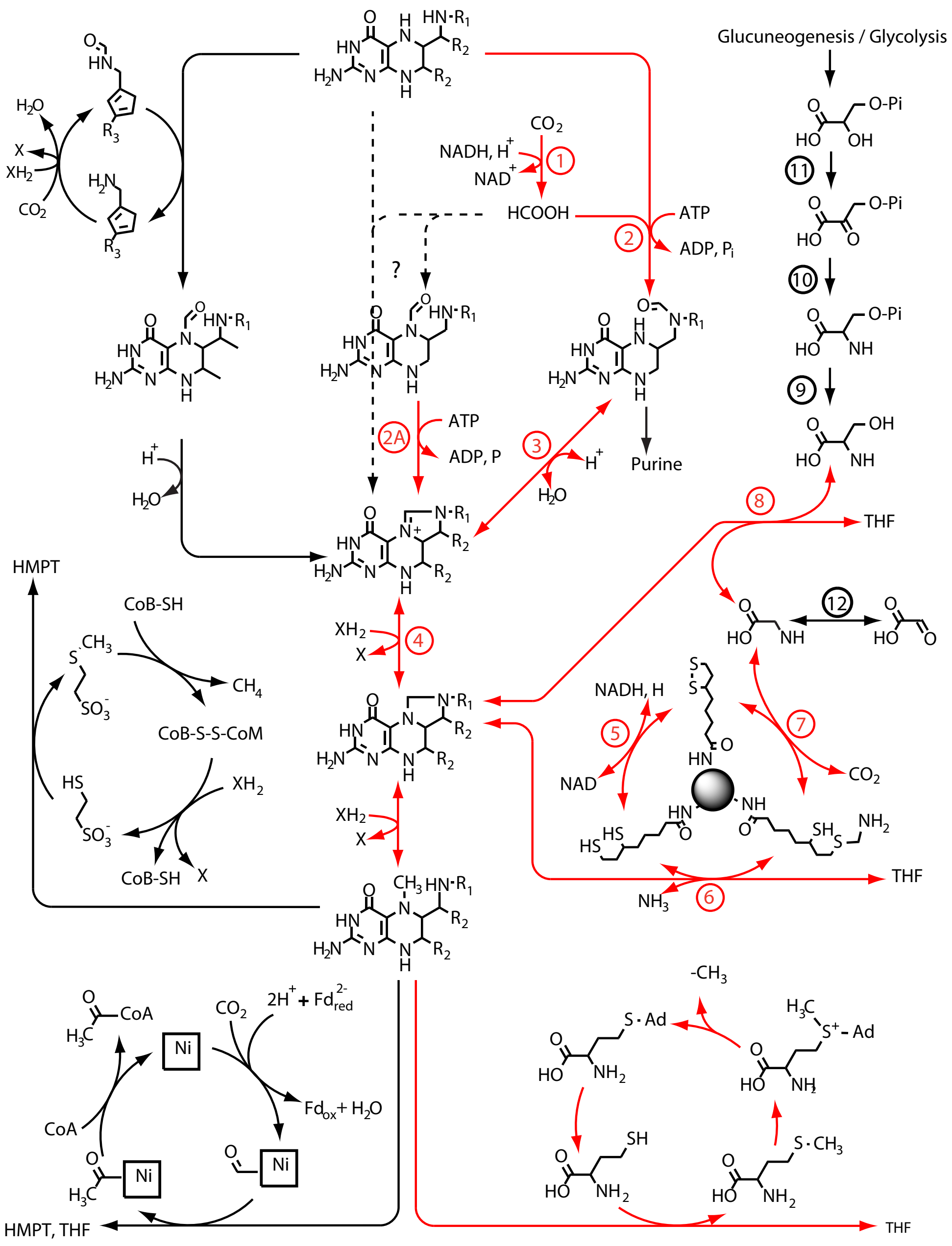

Figure S 1. Summary of C_1 metabolism. Red and numbered reactions represent (as we argue here) the ancestral route to glycine, serine, and methyl-group chemistry. In the Wood-Ljungdahl (WL) pathway these reactions couple to the synthesis of acetyl-CoA as the principal route to carbon-fixation, and to synthesis of either acetate (in acetogens) or methane (in methanogens) for energy metabolism. The difference in substitutions and sidechains (R_1 and R_2) on the basic pterin-moiety between acetogens and methanogens further results in the absence of the lipoic acid-based glycine cycle (reactions 5-8) in the latter. Dashed lines represent suggested alternate routes to formate incorporation through N^5 of THF (see Supporting Discussion). Abbreviations: H_4MPT = tetrahydromethanopterin; THF = tetrahydrofolate; Ni = nickel-center on acetyl-CoA synthase; CoA = coenzyme A; CoB = coenzyme B; CoM = coenzyme M; Fd = ferredoxin; Ad = adenosyl; XH_2 = reductant; P_i = orthophosphate.

Table S 1. Gene profiles for glycine and serine synthesis pathways in deep-branching organisms

Bacteria

Species[a]	Clade[b]	1	2	3	4	5	6	7	8	9	10	11	12	2A
HTH	Aq	•		•	•	•	•	•	•			•		•
TAL	Aq	•		•	•	•	•	•	•			•		•
AAE	Aq	•		•	•	•	•	•	•			•		•[c]
FNO	Th		•	•	•	•	•	•	•			•		
TMA	Th		•	•	•	•	•	•	•			•		
TNE	Th		•	•	•	•	•	•	•			•		
TTE	Fc		•	•	•	•	•	•	•	•		•		
MTA	Fc	•	•	•	•	•	•	•	•			•		•
CAU	Cf		•	•	•	•	•	•	•		•	•		•
CAG	Cf	•	•	•	•	•	•	•	•		•	•		•
NDE	Ns	•	•	•	•	•	•	•	•			•		
TYE	Ns	•	•	•	•	•			•			•		•
CCH	Ch	•		•	•	•	•	•	•	•		•		•
CPH	Ch	•		•	•	•	•	•	•	•		•		•
IPA	Pl	•	•	•	•	•	•	•	•			•	•	•
PMA	Cy			•	•	•	•	•	•			•	•[c]	•
NPU	Cy	•		•	•	•	•	•	•	•		•	•[c]	•

Archaea

Species	Clade	1	2	3	4	5	6	7	8	9	10	11	12	2A
TNE	Tp	•		•	•				•				•	•
PAS	Tp	•		•	•				•	•		•		•
TAG	Dc					•	•	•	•	•		•		•
HBU	Dc	•				•		•	•	•				•
SSO	Sb	•				•	•	•	•			•		•[c]
MSE	Sb	•				•	•	•	•			•		
KCR	Ka	•					•	•	•					•
HMA	Hb	•	•	•	•	•	•	•	•	•		•		•
HBO	Hb	•	•	•	•	•	•	•	•	•		•		•
TAC	Ta	•	•	•	•	•	•	•	•	•				
PAB	Tc	•					•	•	•	•		•		•
MJA	Mc	•							•	•		•		
MBA	Ms	•		•	•				•	•	•	•		
MHU	Mm	•		•	•	•			•	•	•	•		

Notes: [a] For full names see Supporting Methods, [b] Clades: Aq = Aquificales, Th = Thermotogales, Fc = Firmicutes, Cf = Chloroflexi, Ns = Nitrospirae, Ch = Chlorobi, Pl = Planctomycetes, Cy = Cyanobacteria, Tp = Thermoproteales, Dc = Desulfurococcales, Sb = Sulfulobales, Ka = Korarcheota, Hb = Halobacteria, Ta = Thermoplasmatales, Tc = Thermococcales, Mc = Methanococci, Ms = Methanosarcine, Mm = Methanomicrobiales, [c] Gene is missing in annotation, but a UNIPROT BLASTp search identifies differently annotated gene as target when compared to within-clade relatives.

Table S 2. Distribution of alternate direct reductive pathways for deep-branching organisms

Bacteria

	Red(-2)[a]	2A[b]	Red(-2+2A)[c]	Red(-2+2A)/Red(-2)[d]
Aquificae (7)	3	7	3	1.00
Thermotogae (11)	0	0	0	0
Firmicutes (257)				
Bacilli - Bacillales (90)	18	74	17	0.94
Bacilli - Lactobacillales (90)	0	68	0	0
Clostridia (77)	1	65	1	1.00
Deinococcus-Thermus (9)	6	8	6	1.00
Chloroflexi (14)	0	3	0	0
Chlorobi (11)	9	11	9	1.00
Planctomycetes (4)	3	4	3	1.00
Nitrospirae (2)	0	1	0	0
Verrucomicrobia (4)	0	3	0	0
Cyanobacteria (40)	38	26	25	0.66
All (359)	78	270	64	0.82

Archaea

	Red(-2)	2A	Red(-2+2A)	Red(-2+2A)/Red(-2)
Thermoproteales (8)	0	7	0	0
Desulfurococcales (8)	0	6	0	0
Acidilobales (1)	1	1	1	1.00
Sulfulobales (12)	0	12	0	0
Marine I (2)	0	2	0	0
Korarcheota (1)	0	1	0	0
Aciduliprofundum (1)	0	0	0	0
Thermococcales (7)	0	0	0	0
Thermoplasmatales (3)	0	0	0	0
Halobacteria (13)	7	10	7	1.00
Methanococcales (12)	0	0	0	0
Methanobacteriales (6)	0	2	0	0
Methanopyrus (1)	0	1	0	0
Archeoglobus (3)	0	2	0	0
Methanosarcinales (6)	0	0	0	0
Methanomicrobiales (8)	0	6	0	0
All (92)	8	50	8	1.00

Notes: [a] Red(-2) = as in main text, [b] rxn 2A = N^5-formyl-THF cycloligase (see Supplementary Figure 1), [c] number of strains that in the reductive pathway lack only rxn 2 but possess rxn 2A, [d] fraction of strains that lack only rxn 2 but possess rxn 2A.
